# Protective Effects of Celery Juice in Treatments with Doxorubicin

**DOI:** 10.3390/molecules14041627

**Published:** 2009-04-24

**Authors:** Jovanka Kolarovic, Mira Popovic, Momir Mikov, Radoslav Mitic, Ljiljana Gvozdenovic

**Affiliations:** 1 Institute for Child and Youth Health Care of Vojvodina, Department of Hematology/Oncology, Novi Sad, Republic of Serbia; 2 Department of Chemistry, Faculty of Science, University of Novi Sad, 21000 Novi Sad, Republic of Serbia, E-mail: popovic@ih.ns.ac.yu; 3 Medical Faculty and Department of Pharmacology and Toxicology, University of Novi Sad, Republic of Serbia; 4 Institute of Pharmacology, Faculty of Medicine, University of Pristina (Kosovska Mitrovica), Republic of Serbia; 5 Department of Surgery, Faculty of Medicine, University of Novi Sad, 21000 Novi Sad, Republic of Serbia

**Keywords:** Celery juice, Doxorubicin, Reduced glutathione, Lipid peroxidation, Catalase

## Abstract

The aim of this work was to investigate possible protective effect of celery juice in doxorubicin treatment. The following biochemical parameters were determined: content of reduced glutathione, activities of catalase, xanthine oxidase, glutathione peroxidase, peroxidase, and lipid peroxidation intensity in liver homogenate and blood hemolysate. We examined influence of diluted pure celery leaves and roots juices and their combinations with doxorubicine on analyzed biochemical parameters. Celery roots and leaves juices influenced the examined biochemical parameters and showed protective effects when applied with doxorubicine.

## 1. Introduction

Doxorubicin and other anthracyclines are among the most potent chemotherapeutic drugs commonly used in treatment of acute leukemias, lymphomas and different types of solid tumors such as breast, liver and lung cancers. Their clinical use is, however, limited by the risk of severe cardiotoxicity, which can lead to irreversible congestive heart failure. There is increasing evidence that essential components of myocardial energy metabolism are among the highly sensitive and early targets of doxorubicin-induced damage [1,2]. 

Different authors tried to combine potentially cardiotoxic drugs with extracts of medical herbs used as herbal remedy, in attempt to reduce their side effects. Wattanapitayakul *et al*. demonstrated that antioxidants from natural sources may be useful in the protection of doxorubicin induced cardiotoxicity [3]. Israeli authors have evaluated the prophylactic effect of spinach natural antioxidant (NAO) on doxorubicin induced cardiotoxicity and oxidative stress in female mice. Their results indicated beneficial effects of NAO [4]. The cytoprotective herb-derived agent, *Ginko biloba* extract 761 may potentially protect the heart from severe toxicity of doxorubicin [5]. Chinese herbal extracts have been widely used for the treatment of various cancers. Their efficacy was evaluated in pancreatic cancer cell lines. Results revealed that Chinese herbal extracts exhibited significant toxicity in pancreatic cancer cells, mediated via induction of apoptosis [6]. The growth inhibitory activity of doxorubicin or cisplatin, as a single agent, may be modified by combinations of *Phyllanthus emblica* or *Terminalia bellerica* extracts and be synergistically enhanced in some cases [7].

Recent studies however show that combination of drugs and herbal medicines might increase frequency of adverse events, or might change drug metabolism. For example, there are many references showing that interactions between warfarin and extracts of medical herbs could produce both positive and negative synergism [8].

It has been recognized that antineoplastic drugs biotransformed via cytochrome (CYP) 3A4 should not be combined with St John’s Wort (SJW) [9,10,11]. Recently, various St. John's wort constituents were screened for their inhibitory activity on P-glycoprotein (P-gp), and hypericin and hyperforin were identified as potent inhibitors of the P-gp-mediated efflux of the substrates calcein-acetoxymethylester (calcein-AM) and daunorubicin *in vitro* [12]. Furthermore, in vivo, extracts of St. John's wort caused a clinically relevant decrease of the plasma levels of P-gp and CYP3A4 substrates [13,14].

Theanine also helps patients who are undergoing chemotherapy for cancer treatment. Although chemotherapy can provide hope for cancer sufferers, at times it seems that the cancer treatment is almost worse than the disease. Researchers in Japan, where green tea with theanine has been a part of the culture for centuries, found that patients experienced positive results from drinking tea. Patients who added theanine to their diet experienced fewer and less severe side effects from doxorubicin treatments. The doctors in the study felt that drinking green tea could encourage cancer chemotherapy and may improve the quality of life of clinical patients [15,16,17]. Blood sample analysis found that the production of anti-bacterial proteins was up to five times higher in the tea-drinkers, an indicator of a stronger immune response [18].

Luteolin is a naturally occurring compound found in foods including celery, parsley, artichoke leaves, and many others. Doxorubicin caused lipid peroxidation to rise in bone marrow to 5.9 times normal and cardiac rose to 1.5 times normal. Luteolin provided dramatic protection against this drug-induced free radical damage. Bone marrow peroxidation decreased 91% and CPK levels (an indicator of myocardial damage) were normalized by luteolin. Importantly, luteolin did not interfere with the therapeutic effects of doxorubicin [19]. 

Numerous studies show that active principles from plants have multiple effects on metabolism and may alter activity of different drugs. Secondary biomolecules from plants might have diverse effects such as: anti-bacterial and anti-viral activity, anti-inflammatory, anti-angionic, analgesic and anti-allergic effects, hepatoprotective, cytostatic, apoptotic, estrogenic and antiestrogenic properties, antioxidant and prooxidant effects etc. [20,21,22,23,24,25,26]. 

Our previous results have pointed at antioxidant and hepatoprotective effects of celery extracts, as well as influence on pharmacodynamic activities [27,28,29,30]. These findings have led us into examination of influence of celery roots and leaves juices on certain biochemical parameters in doxorubicin treated animals. 

## 2. Results and Discussion

Results obtained by measuring investigated biochemical parameters in liver homogenate are presented in Table 1 as mean with standard deviation. 

**Table 1:** Investigated Biochemical Parameters in Liver Homogenate (mean±SD).

	CON	CR	CL	D	CRD	CLD
**LPx**	0.45±0.06	0.40±0,11	0.22±0.09^a^	0,35±0,06	0.37±0.08	0.34±0,06
**XOD**	2,24±0.19	4.69±1.31^b^	3.35±0.70^a^	2.16±0.14	4.19±0.80^b^	3.34±0.36^a^
**CAT**	0.71±0.13	1.13±0.17^a^	0.95±0.08^a^	0.74±0.24	0.81±0.04	0.96±0.09
**Px**	2.15±0.65	4.54±0.61^c^	3.34±0.13^a^	3.95±0.64^a^	2.98±0.38	2.87±0.80
**GSHPx**	4.52±0.99	6.86±1.13^b^	5.94±0.99^a^	3.97±0.57	5.72±0.40^a^	4.51±0.76
**GSH**	12.20±1.63	19.29±1.02^c^	16.42±3.05^a^	10.72±1.79	9.31±0.86^b^	7.92±1.78^b^

^a^ p<0.05, ^b^ p<0.01, ^c^ p<0.001, t-test, n = 6; x ± SD. Content of GSH is expressed in nmol GSH/mg of protein. Activities of XOD, GSH-Px, GSHR, Px, CAT are expressed in nmol/mg of protein min^-1^. Content of LPx is expressed in nmol malondialdehyde/mg of protein.

The intensity of lipid peroxidaton (LPx) was decreased only by celery leaves juice, while doxorubicin either administered alone, or in combination with celery roots and leaves juices did not show any influence.

Activities of XOD, CAT, Px, and GSHPx were increased by celery roots and leaves juices. Their combination with doxorubicin increased XOD activity, while doxorubicin administered alone did not change activity of this enzyme. Activity of catalase was not influenced by doxorubicin, neither by combination of doxorubicin and celery juices. Peroxidase activity was increased by celery roots and leaves juices and by doxorubicin, while their combination did not influence activity of this enzyme.

Activity of glutathione peroxidase was generally increased, except by celery roots and leaves juices and combination of doxorubicin and celery roots juice. Reduced glutathione content was increased by celery roots and leaves juices and decreased by combination of doxorubicin and both juices, while doxorubicin alone did not show any influence. 

Variability of biochemical parameters analyzed in liver homogenate and blood hemolysate (LPx, XOD, CAT, GSHPx, Px and GSH) among the investigated groups (Table 2) and significance of observed differences was assessed by using one–way ANOVA (one-way analysis of variance) and Tukey Snedecor test F and D values. 

**Table 2:** ANOVA Test for Biochemical Parameters Analyzed in Liver Homogenate.

LPx	CON	CR	CL	CRD	CLD	XOD	CON	CR	CL	CRD	CLD
**CR**	0.05 −					**CR**	2.44 +				
**CL**	0.23 +	0.18 +				**CL**	1.20 +	1.24 +			
**CRD**	0.08 +	0.04 -	0.15 +			**CRD**	1.94 +	0.50 -	0.74 +		
**CLD**	0.11 +	0.06 -	0.12 +	0.03 -		**CLD**	1.09 +	1.35 +	0.11 -	0.85 +	
**D**	0.10 +	0.05 -	0.14 +	0.01 -	0.02 -	**D**	0.09 -	2.53 +	1.29 +	2.03 +	1.19 +
**CAT**	**CON**	**CR**	**CL**	**CRD**	**CLD**	**GSHPx**	**CON**	**CR**	**CL**	**CRD**	**CLD**
**CR**	0.42 +					**CR**	2.35 +				
**CL**	0.11 -	0.31 +				**CL**	1.42 +	0.93 +			
**CRD**	0.24 +	0.18 -	0.14 -			**CRD**	1.20 +	1.15 +	0.22 −		
**CLD**	0.25 +	0.17 -	0.14 -	0.01 -		**CLD**	0.01 −	2.36 +	1.43 +	1.21 +	
**D**	0.04 -	0.38 +	0.14 -	0.21 +	0.22 +	**D**	0.54 −	2.89 +	1.96 +	1.75 +	0.53 −
**Px**	**CON**	**CR**	**CL**	**CRD**	**CLD**	**GSH**	**C0N**	**CR**	**CL**	**CRD**	**CLD**
**CR**	2.39 +					**CR**	7.09 +				
**CL**	1.23 +	1.16 -				**CL**	4.22 +	2.88 +			
**CRD**	0.83 -	1.56 +	0.40 -			**CRD**	2.88 +	9.98 +	7.10 +		
**CLD**	0.72 -	1.67 +	0.51 -	0.11 -		**CLD**	4.28 +	11.37 +	8.50 +	1.39 +	
**D**	1.80 +	0.58 -	0.58 -	0.97 -	1.09 -	**D**	1.48 +	8.58 +	5.70 +	1.40 +	2.80 +

Analyzing activity of CAT, Px, and LPx in liver homogenate by ANOVA, we have found significant difference among seven combinations of groups for catalase (CR/CON, CL/CR, CRD/CON, CLD/CON, D/CR, D/CRD, D/CLD) and five pairs of groups for peroxidase (CR/CON, CL/CON, CRD/CR, CLD/CR, D/CON). The intensity of lipid peroxidaton was significantly different between following group combinations (8): CL/CON, CL/CR, CRD/CON, CRD/CL, CLD/CON, CLD/CL, D/CON, D/CL. Measured xanthine oxidase activity in liver homogenate showed significant difference between 12 pairs of groups, while activity of these enzymes was similar in only three combinations of groups (CRD/CR, CLD/CL, D/CON) (Table 2). Glutathione peroxidase activity was different among 12 combinations of groups (CR/CON, CL/CON,CL/CR, CRD/CON, CRD/CR, CLD/CR, CLD/CL, CLD/CRD, D/CR, D/CL, D/CRD}, while it was similar in four pairs of groups. When glutathione content in liver homogenate was assessed, we have found significant differences among all groups. 

Table 3 shows values of investigated biochemical parameters measured in blood hemolysate expressed as mean with standard deviation. Difference among the control and investigated group was assessed by Student’s t-test. Intensity of lipid peroxidation was decreased in all groups when compared to the control. Activity of enzyme xanthine oxidase was 1.5 fold decreased in groups treated with celery leaves juice and doxorubicin. Activities of catalase and peroxidase in blood hemolysate were decreased by both celery juices and doxorubicin, as well as by their combinations. Activity of glutathione peroxidase and reduced glutathione content were decreased by all agents except for celery roots juice.

**Table 3:** Investigated Biochemical Parameters in Blood Hemolysate (mean±SD).

	CON	CR	CL	D	CRD	CLD
**LPx**	0.27± 0.04	0.22 ± 0.01^a^	0.18 ± 0.03^b^	0.20 ± 0.02 ^b^	0.21± 0.02^a^	0.21± 0.02^a^
**XOD**	2.66 ± 0.19	2.40 ± 0.36	2.34 ± 0.25^a^	2.27 ± 0.21^a^	2.98 ± 0.43	2.53 ± 0.37
**CAT**	2.12 ± 0.22	1.73 ± 0.11^b^	1.83 ± 0.11^b^	1.84 ± 0.12^a^	1.79 ± 0.18^a^	1.76 ± 0.14 ^b^
**Px**	1.86 ± 0.06	1.63 ± 0.16 ^a^	1.40 ± 0.06^c^	1.36 ± 0.13^c^	1.59 ± 0.13 ^b^	1.50 ± 0.24^a^
**GSHPx**	6.63 ± 0.34	6.23 ± 0.44	5.51 ± 0.39^c^	5.12 ± 0.19^c^	5.72 ± 0.72^a^	5.82 ± 0.33^b^
**GSH**	1.47 ± 0.18	1.32 ± 0.11	1.20 ± 0.15^a^	0.87 ± 0.11^c^	1.18 ± 0.11^a^	1.13 ± 0.16^b^

^a^ p<0.05, ^b^ p<0.01, ^c^ p<0.001, t-test, n = 6; x ± SD. Content of GSH is expressed in nmol GSH/mL erythtrocytes. Activities of XOD, GSH-Px, GSHR, Px, CAT are expressed in nmol/mL erythtrocytes min^-1^. Content of LPx is expressed in nmol malondialdehyde/mL erythtrocytes.

When differences between analyzed biochemical parameters (LPx, XOD, CAT, GSHPx,Px and GSH) in blood hemolysate (Table 4) were assessed by one-way analysis of variance (ANOVA) and Tukey Snedecor test F and D values we have obtained the following results: the most expressed was difference in glutathione peroxidase activity (significant among 13 pairs of groups, insignificant only between two combinations of groups – CRD/CL, CLD/CRD), reduced glutathione content (significant between 12 pairs of groups and insignificant between three combinations of groups – CRD/CL, CLD/CL, CLD/CRD) and peroxidase (significant among 12 combinations of groups and insignificant between three combinations – CRD/CR, CLD/CRD, D/CL), while differences in xanthine oxidase activities were concerned significant among eight combinations of groups. Differences among the groups were the least expressed for CAT and intensity of lipid peroxidation. 

**Table 4:** ANOVA Test for Biochemical Parameters Measured in Blood Hemolysate.

LPx	CON	CR	CL	CRD	CLD	XOD	CON	CR	CL	CRD	CLD
**CR**	0.04 +					**CR**	0.26 +				
**CL**	0.08 +	0.04 +				**CL**	0.33 +	0.06 -			
**CRD**	0.06 +	0.02 -	0.02 -			**CRD**	0.32 +	0.58 +	0.65 +		
**CLD**	0.05 +	0.01 -	0.03 +	0.01 -		**CLD**	0.13 -	0.13 -	0.19 -	0.45 +	
**D**	0.06 +	0.02 -	0.02 -	0.00 -	0.01 -	**D**	0.39 -	0.13 -	0.06 -	0.71 +	0.26 +
**CAT**	**CON**	**CR**	**CL**	**CRD**	**CLD**	**GSHPx**	**CON**	**CR**	**CL**	**CRD**	**CLD**
**CR**	0.39 +					**CR**	0.40 +				
**CL**	0.29_+_	0.10 -				**CL**	1.12 +	0.72 +			
**CRD**	0.33 +	0.06 -	0.04 -			**CRD**	0.91 +	0.51 +	0.21 −		
**CLD**	0.36 +	0.03 -	0.07 -	0.03 -		**CLD**	0.81 +	0.41 +	0.31 +	0.10 -	
**D**	0.28 +	0.11 -	0.02 -	0.05 -	0.08 -	**D**	1.50 +	1.11 +	0.39 +	0.59 +	0.69 +
**Px**	**CON**	**CR**	**CL**	**CRD**	**CLD**	**GSH**	**C0N**	**CR**	**CL**	**CRD**	**CLD**
**CR**	0.23 +					**CR**	0.14 +				
**CL**	0.46 +	0.23 +				**CL**	0.26 +	0.12 +			
**CRD**	0.27 +	0.04 -	0.19 +			**CRD**	0.28 +	0.14 +	0.02 -		
**CLD**	0.35 +	0.13 +	0.10 +	0.08 -		**CLD**	0.34 +	0.19 +	0.07 -	0.05 -	
**D**	0.50 +	0.27 +	0.04 -	0.22 +	0.14 +	**D**	0.59 +	0.45 +	0.33 +	0.31 +	0.26 +

Results of the ANOVA test are represented for the differences between groups for the confidence level p<0.05; + statistically significant p < 0.05; - statistically nonsignificant p > 0.05; LPx: F = 2.95; p<0.05; D=0.02; XOD: F = 3.63; p<0.01; D = 0.20; CAT: F = 1.63; p>0.05; D = 0.16; Px: F= 9.95; p<0.001; D=0.09; GSHPx: F= 9.78, p<0.001; D = 0.29; GSH: F= 18.51, p<0.001; D = 0.09.

Celery leaves juice applied alone or with doxorubicin has decreased intensity of lipid peroxidation which is considered to be one of oxidative stress markers. We might conclude that celery leaves juice have shown protective effect when compared to celery root juice that did not change this parameter.

Celery roots and leaves juices applied alone or with doxorubicin, increased XOD activity. XOD was the first enzyme approved to be prooxidant by producing large amounts of H_2_O_2_ and O_2_^•–^. This is especially true for the processes of tissue ischemia and reperfusion which are followed by subsequent tissue damage. Our results do not suggest that celery roots and leaves juices show protective effect. This result is in correlation with increased activity of CAT, Px and GSHPx which indicates increased content of H_2_O_2_. Popovic *et al*. [30] in their paper examining influence of different extracts of celery leaves, reported that three out of five investigated extracts have decreased XOD activity (Et_2_O, CHCl_3_ and nBuOH), while other two extracts (EtOAc an H_2_O) have increased. Different influence of above mentioned celery leaves extracts is probably caused by their different chemical composition. 

Luteolin and quercentin, flavonoids and acknowledged antioxidants, could be found in many plants including celery. Most resources do not suggest major influence of luteolin and quercetin on doxorubicin metabolism, but it does not mean that their combination does not alter investigated biochemical parameters [31]. If we analyze data from Table 3**,** activities of XOD, CAT, Px and GSPHx are either lower or similar to the control value. Intensity of lipid peroxidation is decreased as well as reduced glutathione content. 

In our experiment liver was the target organ. We assume that the increased activity of enzymes measured in liver homogenate (XOD, CAT, Px, GSPHx) is not prooxidative, and that moreover, such enhanced activity protects doxorubicin from possible oxidation, and therefore prevents generation of toxic metabolites.

Lakhanpal and Rai [22] suggested that quercetin may enhance the effect of doxorubicin. Quercetin seems to inhibit XOD, thereby resulting in decreased oxidative injury. 

We know that doxorubicin produces clinically restorative responses in numerous human cancers, but its cardiotoxicity has limited its usefulness. Israeli researchers evaluated the prophylactic effect of spinach natural antioxidant on doxorubicin-induced cardiotoxicity and oxidative stress in female Balb/c mice. They found that pretreatment with spinach natural antioxidant before doxorubicin administration decreased catalase and increased superoxide dismutase activities compared to the doxorubicin group [3]. Obtained results suggest that both celery juices influence investigated parameters, because their chemical profile is similar. In our paper [30] methanol extracts of celery roots and leaves showed similar effects on OH radical production and intensity of lipid peroxidation in *in vitro* experiments. 

Single pretreatment of mice with celery increased and prolonged the analgesic effect (hot-plate results) of aminopyrine, statistical significance being attained only in the interaction with acetaminophen [27]. Other authors reported that celery and parsley extracts did not exhibit significant effects on the induction time in mice treated with ketamine. On the other hand, both plants caused prolongation of sleeping time in animals [29]. 

Other study found that treatment with celery extracts showed both positive and negative synergism with CCl_4_, inducing or suppressing its activity. The results obtained indicate that no significant relationship can be established between the action of celery extracts and prevention of oxidative stress. Based on the experimental results, the strongest protective effect was shown by the *n*-BuOH extract [30]. 

Such “unprotective” results are in collision with “protective” effect, because intensity of lipid peroxidation was decreased, while reduced glutathione content was increased. In order to achieve better understanding of celery roots and leaves juices influence on doxorubicin metabolism, we propose additional experiments. We suggest investigating the influence of combination of doxorubicin and different celery extracts or isolated “active principles” from celery and doxorubicin on antioxidant systems.

## 3. Experimental

### 3.1. General

Whole fresh plants were used in this study. Celery leaves were separated from roots. Fresh leaves were ground in a blender and 5% solution (v/v) was prepared by diluting pure juice with distilled water. The same blender was used for grounding celery roots and 5% (v/v) solution was prepared by diluting with distilled water. 

This investigation was conducted on sexually mature male Whistar laboratory rats, with an average body weight of 250-300 grams and ages up to 3 months. Animal care and all experimental procedures were conducted in accordance with the *Guide for the Care and Use of Laboratory Animal Resources*, edited by Commission of Life Sciences, National Research Council, Male and female Hanover National Medical Institute (Hann NMRI). Rats were bred in the vivarium at the Department of Pharmacology, Toxicology and Clinical Pharmacology, Medical Faculty, University of Novi Sad, Serbia. Animals were kept in standard plexiglass cages at constant room temperature 21±1 ^0^C ºC and humidity 55%±1.5%, with circadian rhythm (day/night). They were fed standard laboratory rat feed, produced by the Veterinary Institute in Zemun. Animals were given free access to food and fluid (water or fresh celery roots or leaves juices). Rats were divided into 7 experimental groups comprising 6 animals each. Average dose of doxorubicin was selected on the basis of common human dosage and Clark's formula.

### 3.2. Animal treatment

Group I was the control (CON), animals from this group were drinking only water *ad libitum*; animals from group II (CR) were instead of water drinking only celery roots juice; group III (CL) was given only celery leaves juice; animals from group IV (CRD) were exclusively drinking celery roots juice and received doxorubicin (1.5 mg/kg) intraperitoneally (ip) 4 times in 14 days (on days 1, 5, 9, 13); group V (CLD) was drinking only celery leaves juice and was treated with doxorubicin (1.5 mg/kg ip) on days 1, 5, 9, 13; group VI (D) was drinking water and received doxorubicin (1.5 mg/kg ip) on days 1, 5, 9, 13.

Doxorubicin treated groups received the drug every fourth day, while the other groups at the same time, instead of doxorubicin received 0.9% sodium chloride (10 mL/kg body weight) intraperitoneally. Intervals between two concomitant applications of doxorubicin resemble the most frequent treatment schedules in human medicine. 

On the day 17 (3 days after the last dose of doxorubicin) all animals were sacrificed in urethan anesthesia. Blood samples were collected and livers were removed and homogenized.

### 3.3. Biochemical assays

We have measured examined biochemical parameters in blood hemolysate and liver homogenate. Liver was homogenized in a Potter homogenizer with TRIS-HCl/sucrose in a ratio of 1:3 at 4^o^C. Obtained homogenate was filtered. The following biochemical parameters were analyzed in blood hemolysate and liver homogenate: extent of lipid peroxidation (LPx) was determined after Buege and Aust [32], peroxidase (Px) activity was measured after Simon *et al*. [33], catalase activity (CAT) after Beers and Sizer [34]. Glutathione peroxidase (GSH-Px) activity was evaluated as described by Chin *et al*. [35], xanthine oxidase (XOD) after Bergmayer [36], content of reduced glutathione (GSH) in blood after Beuthler *et al.* [37] and in the liver after Kapetanoviç and Mieyal [38]. The total protein content in liver was determined after Gornall *et al*. [39].

### 3.4. Chemicals

Doxorubicin was obtained from Pharmacia & Upjohn Inc (New Haven, CT, USA), guaiacol, xanthine, cumene hydroperoxide were obtained from Sigma-Aldrich (St. Louis, Missouri, USA), DTNB and reduced glutathione was obtained from Merck (Darmstadt, Germany). All chemicals used were of analytical grade.

### 3.5. Statistical analysis

Results of biochemical analyses are presented as the mean value±standard deviation (SD). The difference between control and test groups was analyzed using the Student t-test (significant difference at p ± 0.05 confidence level). Using one-way ANOVA (one-way analysis of variance) and Tukey Snedecor test F and D values, parameters which present the total variability values and significance of differences observed between groups, were assessed. 
